# In vitro Anti-Tumor Effects of Statins on Head and Neck Squamous Cell Carcinoma: A Systematic Review

**DOI:** 10.1371/journal.pone.0130476

**Published:** 2015-06-22

**Authors:** Ludmila Madeira Cardoso Pavan, Daniela Fortunato Rêgo, Silvia Taveira Elias, Graziela De Luca Canto, Eliete Neves Silva Guerra

**Affiliations:** 1 Laboratory of Oral Histopathology, Health Sciences Faculty, University of Brasília, Brasília, Brazil; 2 Department of Dentistry, Federal University of Santa Catarina, Florianópolis, Brazil, and University of Alberta, Edmonton, Canada; University of Manitoba, CANADA

## Abstract

**Background:**

Statins are commonly used against arteriosclerotic disease, but recent retrospective analyses have suggested that statins also prevent cancer. The aim of this systematic review is to verify the vitro anti-tumor effects of statins on head and neck squamous cell carcinoma.

**Methods:**

Studies were gathered by searching Cochrane, MEDLINE, EMBASE, LILACS, and PubMed, up until May 9, 2015, with no time or language restrictions. Only in vitro studies that discuss the effect of statins on head and neck carcinoma were selected.

**Results:**

Of 153 identified papers, 14 studies met the inclusion criteria. These studies demonstrated that statins had a significant effect on head and neck squamous cell carcinoma cell lines and influenced cell viability, cell cycle, cell death, and protein expression levels involved in pathways of carcinogenesis, which corroborates with the potential in vitro anti-tumor effects. It provides highlights about the biological mechanisms of statins used alone or associated with traditional therapy for cancer.

**Conclusions:**

Though there are few studies on the topic, currently available evidence suggests that statins shows that preclinical experiments supports the potentiality of statin as an adjuvant agent in chemotherapy and/or radiotherapy approaches routinely used in the management of HNSCC and should undergo further clinical assessment.

## Introduction

The statin family of drugs is known worldwide as a safe and effective therapeutic agent for the treatment of arteriosclerotic cardiovascular disease [1]. Statins prevent the synthesis of cholesterol in the liver and reduce the levels of low-density lipoprotein (LDL), lipids, and blood cholesterol, which in turn significantly increases the survival of patients [2]. Statins are potential inhibitors of 3-hydroxy-3-methylglutaryl reductase A (HMG-CoA), an enzyme involved in the mevalonate pathway [2, 3, 4]. The use of HMG-CoA reductase inhibitors to inhibit the rate-limiting step of the mevalonate pathway results in decreased levels of mevalonate and its downstream products; this may significantly influence many critical cellular functions [5]. Statins have the potential to exert pleiotropic cellular effects and can inhibit the growth, invasion, metastasis, cellular proliferation and differentiation, and cell cycle regulation of tumor cells [6, 7, 8]. These drugs also induce apoptosis, and when used alone can stabilize the disease especially in squamous cell carcinoma [9,10].

Statins have demonstrated an ability to enable different tumor induction pathways, mediated by metabolic stress that regulates tumor cell apoptosis. By inhibiting the mevalonate pathway, statins can inhibit the function of epidermal growth receptor (EGFR), which inhibits the mammalian target of rapamycin (mTOR) cascade and the phosphoinositide 3-kinase (P13K/AKT) pathway [8,11]. Additionally, they regulate translation of mRNA that encodes pro-oncogene proteins, thereby inhibiting both proliferation and survival of malignant cells [12]. Oral and pharyngeal cancers are the sixth most common form of cancer in the world. The risk of developing oral cancer increases with age, and the majority of cases occur in people aged 50 or over. In most countries, five-year survival rates for cancers of the tongue, oral cavity, and oropharynx are around 50%, although many patients who are successfully treated for oral cancer have to cope with the devastating consequences of their treatment [13]. Thus, the concept of using statin as a chemopreventive agent to control carcinogenesis is promising [14, 15]. Recent retrospective analyses have suggested that statins also prevent cancer [3, 6, 7, 8, 9, 10, 11]. Therefore, the aim of this systematic review is to use the available literature to verify the vitro anti-tumor effects of statins on head and neck squamous cell carcinoma.

## Methods

### Protocol and registration

The Preferred Reporting Items for Systematic Reviews and Meta-Analysis (PRISMA) Checklist was followed in this systematic review [16]. We did not register a protocol.

### Eligibility criteria

#### Inclusion criteria

We selected only articles that compared the effect of statins to control substances in the context of squamous cell carcinoma treatment. The cell lines used should be from head and neck squamous cell carcinoma (HNSCC), such as cells from lip and/or oral cavity, pharynx, larynx, nasal cavity, and paranasal sinuses [17]. All of the included papers were in vitro or in vivo animal studies. The PICOS (population, intervention, comparison, outcome, study design) format was adapted to define a clinical question with the following inclusion criteria:

Population: Cells or animal.

Intervention: Statin use for prevention or treatment of HNSCC.

Comparison: Cells or animals that did not receive statin treatment but have received a control treatment.

Outcome: Cell viability, apoptosis, cell cycle arrest, and regulation of protein expression levels.

Study Design: Randomized or non-randomized controlled trials (in vivo animal studies) or studies with comparable or no comparable baseline (in vitro studies).

#### Exclusion criteria

We have excluded: 1) Studies with different targets, such as ones that did not use statins to treat cancer or did not verify the association between statins and HNSCC; 2) Reviews of the literature, letters, case reports, personal opinions, conference abstracts, and book chapters; 3) Clinical studies.

### Information sources and search strategies

A criteria search was performed using the following electronic bibliographic databases: Cochrane, MEDLINE, EMBASE, LILACS and PubMed. More information regarding the search strategies is provided in S1 Table. The reference list was checked at the end of search. We conducted all searches across all databases from the earliest available date up May 9, 2105. We curated the references manually and removed duplicate hits.

#### Study selection

We selected articles in two phases. In phase one, two authors (L.M.C.P, E.N.S.G) independently reviewed the titles and abstracts of all the references. The articles that appeared to meet the inclusion criteria based on their abstract were selected and collected. In phase two, two authors (L.M.C.P and E.N.S.G) read all the full-text articles and excluded those which were not in agreement with the inclusion criteria. The same two authors independently reviewed all full-text articles. Any disagreement between the authors in the first and second phases was resolved by means of discussion and mutual agreement. When they did not reach a consensus, a third author (S.T.E) intervened to make a final decision.

#### Data collection process and data items

One author (L.M.C.P) collected the required information from the selected articles such as authors, year of publication, country, study design, assays, treatment used, results, and main conclusions (Table 1). A second author (E.N.S.) crosschecked all of the retrieved information. Again, any disagreement was resolved by means of discussion and mutual agreement between the two authors. When they did not reach a consensus, a third author (S.T.E.) intervened to make a final decision.

**Table 1:** Summary of descriptive characteristics of included articles (n = 14).

Author and year	Country	Study design	Methods: Assays	Methods: Cell line/ Origen	Methods: Treatment	Results	Main conclusion	Clinical application
Dayekh et al, 2014 [23]	Canada	In vitro	MTT assay; Flow cytometry; Western blot; Fluorescence microscopy; Transcriptome analysis (RNAseq); RT PCR; Ex vivo tumor analysis	SCC9; SCC25; GM-38	Lovastatin; Erlotinib; Monensin + Erlotinib; Monensin + Lovastatin	Monensin in combination with lovastatin or erlotinib resulted in a marked decrease in viability in a dose-dependent manner in SCC9 and SCC25 cells (90% of cell death, in 48 hours, in both treatment and cells compared with lovastatin or erlotinib alone). Combination of Monensin with erlotinib increased apoptosis in SCC25 cells (38.7% apoptosis vs. 3.4% control). Monensin treatments enhanced the inhibitory effects of erlotinib on ligand-induced EGFR activation as measured by pEGFR levels.	Monensin enhanced the cytotoxic action of both lovastatin and erlotinib treatments in SCC9 an SCC25 cells. Monensin mimics the inhibitory effects of lovastatin on EGFR activity.	1
Dimitroulakos et al, 2001 [20]	Canada	In vitro	MTT assay; Flow cytometry; Mass spectroscopy; HPLC	SCC4; SCC25; SCC15; SCC9; FADU; CAL27	Lovastatin;Mevalonate;Ethanol (Control)	Lovastatin induces apoptosis and cytotoxicity. HNSCC cells lines had about 60 to 80% of death.	Apoptosis induced by lovastatin is mediated by the open ring activated form of the mevalonate drug that targets HMG- CoA reductase. Taken together, these drugs may represent a therapeutic approach.	1
Dimitroulakos et al, 2002 [9]	Canada	In vitro	cDNA expression; Microarray blots; Luciferase Promoter activity assay; MTT assay; Western blot	SCC9; SCC25; SIHA; Cos-7	Lovastatin; Ethanol (Control)	Lovastatin promotes cell death, block the production of specific mevalonate derivates and induce cytotoxicity in HNSCC and CC cells. RhoA is a gene that is potentially regulated by lovastatin. Causes cytotoxicity in HNSCC and CC cells. The lovastatin treatment promotes 50% of cell death, with a dose dependent manner.	The depletion of mevalonate metabolites, particularly GGPP, can be a potential mediator of lovastatin-induced apoptosis in HNSCC and CC cells.	1
Gabryś et al, 2008 [25]	Germany	In vitro and In vivo animal	MTT assay; Flow cytometry; Western blot; Tumor transplantation; Irradiation, follow-up, and tumor growth delay	U87MG; FaDu	Lovastatin; Lovastatin + Radiation; Ethanol (Control)	Lovastatin inhibits cell proliferation, induces apoptosis and decreases tumor volume over time but does not increase growth delay after irradiation of U87MG tumors with 20 Gy (gray). No significant differences were observed after lovastatin plus irradiation treatment, compared with irradiation alone (p<0.001).	Lovastatin did not improve the effects of radiation on in vivo tumors.	1
Islam et al, 2013 [7]	USA	In vitro and In vivo animal	Motility assay; Invasion assay; Confocal microscopy; Cell proliferation assay; In vitro clonogenic survival assay; Western blot	UM- SCC-1; UM-SCC-47	Atorvastatin; Ethanol (Control)	Atorvastatin decreases the expression of active RhoC; Reduces cell invasion in HNSCC cell lines (p<0.05); Decreases cell motility, colony formation and proliferation when compared to control in a dose-response effect (p<0.05); Results a small decrease in tumor growth in treated mice.	Atorvastatin treatment decreases cell invasion, migration and colony formation in HNSCC cell lines. This medicine also decreases the membrane fraction of RhoC and limits the activation of ERK1/2 and STAT3 signaling cascades. In an in vivo model, RhoC inhibition resulted in an inhibition of metastases.	1
Llobet, et al, 2014 [26]	Spain	In vitro and In vivo animal	Wound healing assay; Cell proliferation assay; Clonogenic assay; XRT and pharmacological treatments; Xenografts and in vivo treatments; Western blot	A431; FADU	Cetuximab; Simvastatin; Radiation; Radiation + Simvastatin; Cetuximab + Simvasatin; Radiation + Cetuximab; Radiation + Cetuximab+ Simvastatin; DMSO (Control)	Simvasatin decrease wound healing, cell proliferation, and clonogenic survival of cells treated (p<0.05 vs. control in 72 hours), slowed the growth of FADU xenografts treated (p<0.05 vs. control), induced apoptosis (p<0.05 vs. radiation +cetuximab), when used with cetuximab and radiaton. Simvastatin has a weak effect on the activation of phosphorylated AKT and phosphorylated STAT3 and lacked of a dose-response inhibitory effect compared to ERK1/2 protein.	Simvastatin may enhance antitumor response of concomitant radiation and cetuximab. The authors concluded evidence that supports further basic and clinical investigation of simvastatin in HNSCC disease.	1
Ma et al, 2012 [8]	Canada	In vitro	MTT assay; Western blotting; Imunno-fluorescence; ADP/ATP ratio determination; Reactive Oxygen Species quantitation assay	SCC9; SCC 25; HeLa; A549; MEFs/L; KB1	Lovastatin; Metformin; Gefitinib; Lovastatin + Gefitinib; Ethanol (Control)	Lovastatin induces LKB1 and AMPK activation in SCC cells, affects ATP Levels in SCC cells and can enhance the cytotoxic effects of Gefitinib in LKB1 deficient tumor cells.	Lovastatin induces multiple metabolic stress pathways, including the LKB1/AMPK pathway, that enhances lovastatin’s ability to synergize with gefitinib in SCC cells.	1
Mantha et al, 2003 [21]	Canada	In vitro	MTT assay; Flow cytometry; Western blotting	SCC9; SCC25; MCF-7	Lovastatin; Cisplatin; 5-FU; Paclitaxel; Carboplatin; Oxaliplatin	Lovastatin induces apoptosis and reduces SCC9 and SCC25 cell viability. MTT assay–SCC9 35% cell viability in 50μM; SCC25 30% cell viability in 50 μM. Flow cytometric–SCC25 –Lovastatin induces apoptosis 20,4% (10μM) and 34% (50μM). Combinations of either high or low concentration of cisplatin or 5-FU did not potentiate the apoptotic effects of Lovastatin in SCC25 cells. Lovastatin may target EGFR function when the SCC9 and SCC25 cell were treated.	Lovastatin may target the EGFR pathway in HNSCC cells and can potentiate the apoptosis.	1
Mantha et al, 2005 [11]	Canada	In vitro	MTT assay; Flow cytometry; Western blotting	SCC9; SCC25; FADU; CAL27; MCF-7	Lovastatin; Pravastatin; Gefitinib; Lovastatin + Gefitinib	Lovastatin may target the EGFR pathway in HNSCC cells and can induce apoptosis. Lovastatin and Gefitinib together can target the EGFR signaling pathway. The combined lovastatin and Gefitinib treatment enhances cytotoxic and apoptotic response in SCC9 cells.	The combination of statins and EGFR tyrosine kinase inhibitors is an attractive therapeutic approach in HNSCC.	1
Niknejad et al, 2007 [22]	Canada	In vitro	Western blot; Real Time RT-PCR; MTT assay; Flow cytometry; Fluorogenic proteosome assay	SCC25; HeLa; 293T; MEFs WT CHOP-/-	Lovastatin; Lovastain + Mevalonate;Ethanol (Control)	Lovastatin induces phosphorylation of Ie2Fa. ATF3 expression is regulated by lovastatin treatment. CHOP expression is induced by lovastatin in SCC25 cells (p<0.05).	Lovastatin activates ISR in HNSCC cells and controls cell cycle and apoptotic signaling.	1
Nikejad et al, 2014 [6]	Canada	In vitro	MTT assay; Flow cytometry; Western blot; Real time RT-PCR; shRNAs- expressing cells; Mitochondrial membrane permeability assay; Generation of stable eIF2; ATF3 promoter activity; Ex vivo tumors analysis; Caspase 3 activity.	MCF-7; SCC9; SCC25; HeLa; MEFs ATF3 -/-	Lovastatin; Mevalonate; Salubrinal; Lovastatin + Salubrinal; Ethanol (Control)	SCC 25 and HeLa are sensitive to lovastatin treatment–induced cytotoxicity (80% of cell death). There is a decreasing cyclin D1 level in the SCC9 cells treated with Lovastatin, along with specific phosphorylation of both LKB1 and AMPK. This cell line showed a dose-dependent induction of ATF3 with Lovastatin treatment. SCC 25 resulted in a significant induction of ATF3 in a dose-dependent manner, and a pronounced pre-G1 apoptotic peak at 10 and 25μM; Salubrinal enhances lovastatin induced ATF3 expression and cytotoxicity.	Combining Lovastatin with Salubrinal enhanced ATF3 expression and induced synergistic cytotoxicity in SCC cells.	1
Pioche- Durieu et al, 2005 [24]	France	In vitro	Flotation assay; Western blot; Imunoflorescence; Electron microscopy	NPC; C15; C17	Simvastatin; DMSO (Control)	Simvastatin is cytotoxic to NPC cells, decreases cellular viability, and changes cellular morphology. The data not showed the account of viability cell in numbers or percentage.	There was no significance effect of Simvastatin on the distribution of LMP1 and Galectin in NPC cell rafts.	1
Takeda et al, 2006 [27]	USA	In vitro	MTT assay; Flow cytometry; RT PCR; Matrigel invasion assay; Microarray hybridization; Alexa fluorescein-labeled phalloidin staining of actin filaments	Tu-167; JMAR	Simvastatin; DMSO (Control)	The drug inhibits cell proliferation (p<0.01) and migration into the extracellular matrix and invasive activity. It can control the cell cycle and apoptotic signaling (p<0.01).	Simvastatin treatment of HNSCC cell lines leads to the inhibition of cell growth and invasiveness, along with cell cycle arrest and these effects are associated with down regulated of ß1- integrin and ERK signaling. Simvastatin has the potential to be effective for the prevention of growth and metastasis of cancer.	1
Wang et al, 2011 [28]	USA	In vitro	MTT assay; Caspase-3 activity; Cell colony formation	CCL-30	Statin; Mevalonate; Cisplatin; Statin + Cisplatin; Mevalonate + Statin; DMSO (Control)	Statins cause significant decrease in cell proliferation (p < 0.01 vs. control) and viability in NPC, induce changes in cellular morphology, decreases colony formation and promote loss of cellular sphere formation.	The addition of statins to cisplatin causes significant impact on cell survival, better than statin alone p < 0.05 vs. control–the medicine alone. The statins can potentially act as chemo-adjuvant agents in the treatment of NPC.	1

The statin clinical application was classified as (1) potential effect in HNSCC treatment; (2) inconclusive, and (3) evidence not supportive as a drug to HNSCC treatment. Abbreviations: U87MG—Human primary glioblastoma cell line; Gy–Gray; ISR—Integrated stress response; HNSCC—Head and neck squamous cell carcinomas; CC—Cervical carcinoma; EGFR—Epidermal growth factor receptor; LKB1- The Liver Kinase B1; AMPK- AMP-Activated Protein Kinase; RhoC—a GTPase belonging to the Ras superfamily; SCC—Squamous cell carcinomas; EGF—Epidermal growth factor; ATF3- activation of transcription factor; mRNA- Messenger RNA; 5-FU 5-fluorouracil; NPC–Nasopharyngeal Carcinoma; LMP1 –latent membrane protein 1; NPC.- Nasopharyngeal Cancer; GGPP- Geranyl pyrosphosphate; ERK- Extracellular-signal-regulated kinases; RT PCR- Reverse transcription polymerase chain reaction; UM- SCC-1—Squamous cell carcinoma cell lines derived from floor of the mouth; UM-SCC-47—Squamous cell carcinoma cell lines derived from tongue; DMSO—Dimethyl sulfoxide; SCC9 (Homo sapiens tongue squamous cell carcinoma); SCC25 (Homo sapiens tongue squamous cell carcinoma); HeLa (cervical carcinoma); A549 (lung carcinoma); MEFs/LKB1 -/- (Murine Embryonic Fibroblast); CCL-30 (Nasopharyngeal carcinoma cells); 293T (Human Embryonic Kidney); Tu167 (Squamous cell carcinoma cell lines derived from floor of the mouth); JMAR (Squamous cell carcinoma cell lines derived from floor of the mouth); CAL27 (Oral Squamous Cell Carcinoma); SIHA (Cervical Carcinoma); Cos-7 (monkey Kidney Cell Line); SCC4 (Oral epithelial cell lines); SCC15 (Oral epithelial cell lines); C15 (Nasopharyngeal Carcinoma Cells); C17 (Nasopharyngeal Carcinoma Cells); A431 cell (epidermoid carcinoma); GM-38 (Diploid fibroblasts cell line).

#### Risk of bias in individual studies

The authors methodically appraised all of the selected studies according to the GRADE method to judge the quality of evidence [18]. Two authors (L.M.C.P and E.N.S.G) categorized the included articles as “high”, “moderate”, “low” or “very low” quality, according to their analysis of each study. When they did not reach a consensus regarding the quality, a third author (S.T.E.) intervened to make a final decision. We classified studies with comparable baselines, which were compared as a Randomized Controlled Study (RCT), according to the work of Xiao et al. published in 2013 [19].

#### Summary measures

Cell viability, apoptosis, and cell cycle arrest after statin treatment and regulation of protein expression level in HNSCC were the main evaluated outcomes.

#### Synthesis of results

A meta-analysis was planned, since the data from the included studies was considered relatively homogeneous.

#### Risk of bias across studies

Only to be applied if meta-analysis was possible.

## Results

### Study selection

**Fig 1:**
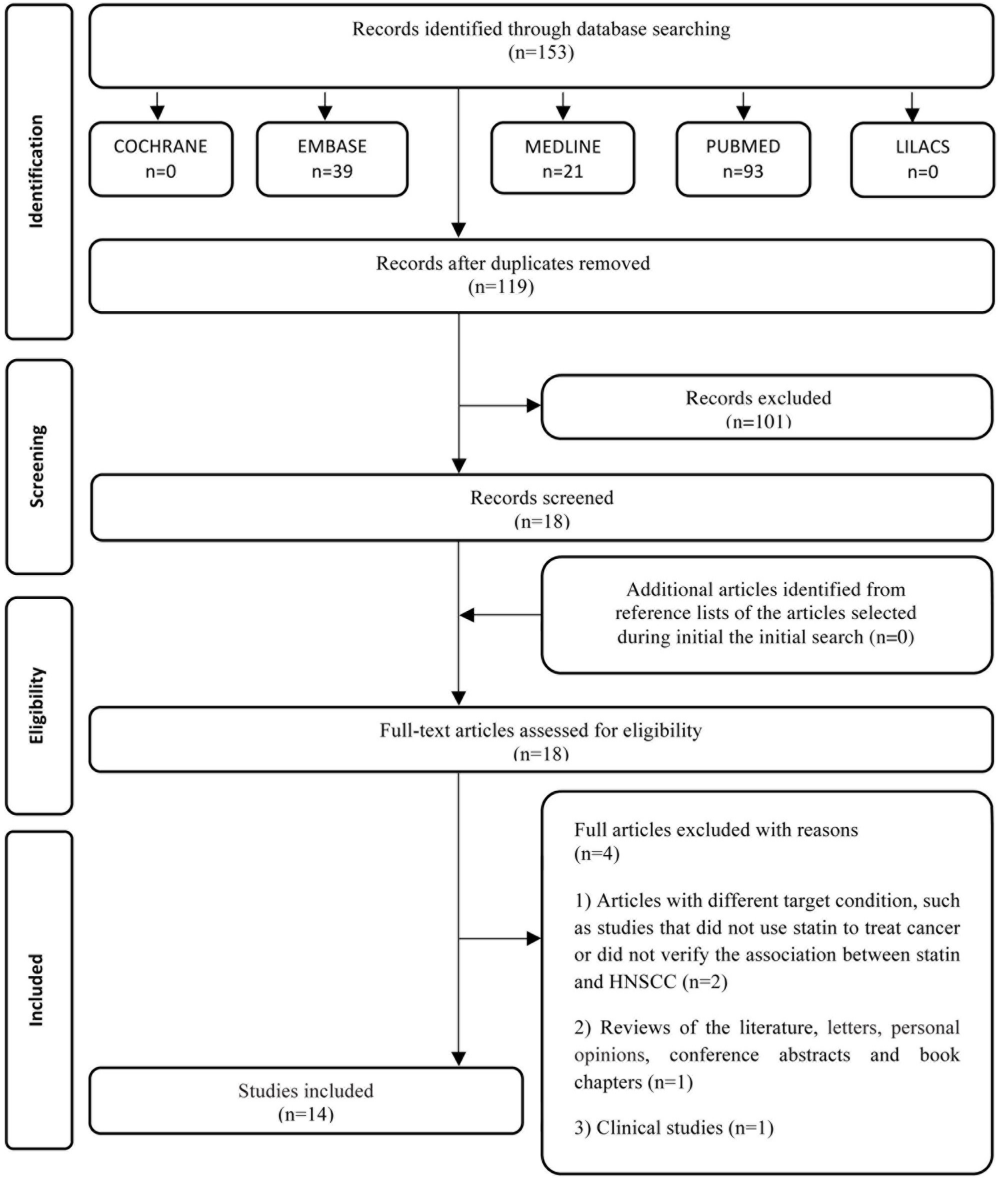
Flow Diagram of literature search and selection criteria adapted from PRISMA [16].

In phase one, 153 papers were selected from five electronic databases. After the duplicates were removed, only 119 different citations remained. Subsequently, we conducted a comprehensive evaluation of the abstracts and excluded 101, resulting in 18 articles at the end of phase one. We did not identify any additional studies from the reference lists of these studies. Subsequently, we retrieved the 18 articles, and following a review of the texts, four more articles were discarded (S2 Table). A flow chart detailing the process of identification, inclusion, and exclusion of studies is shown in Fig 1.

### Study characteristics

The studies were published from 2001 to 2014, and all of them were in English. They were conducted in five different countries: Canada [6, 8, 9, 11, 20–23], France [24], Germany [25], Spain [26] and the United States of America [7, 27, 28]. All of the selected studies were in vitro [6–9, 11, 20–27]; however, three of them also involved in vivo experiments [7, 25, 26]. A summary of the studies is presented in Table 1. A summary of interventions to test HNSCC cell viability in cultured cells is shown in Table 2.

**Table 2:** Interventions used to test head and neck carcinoma cell lines viability in cell culture.

	P	I			C	O	S	%
Studies	Cells	Interventions	Treatment time (hours)	Dose				
Dayekh et al, 2014 [23]	SCC9; SCC25; GM-38	Lovastatin; Erlotinib; Monensin + Erlotinib; Monensin + Lovastatin	24–48	0–10 μM Lovastatin; 0–10 μM Erlotinib; 0–2 μM Monensin	√	√	√	1
Dimitroulakos et al, 2001 [20]	SCC4; SCC25; SCC15; SCC9; FADU; CAL27	Lovastatin; Ethanol (Control)	24–48	1–100 μM	√	√	√	1
Dimitroulakoset al, 2002 [9]	SCC9; SCC25; SIHA; Cos-7	Lovastatin; Ethanol (Control)	24–48	0–100 μM	√	√	√	1
Gabryś et al, 2008 [25]	U87MG; Fadu	Lovastatin; Lovastatin + Radiation; Ethanol (Control)	24–48–72	0–50 μM Lovastatin	√	√	√	1
Islam et al, 2013 [7]	SCC-1; SCC-47	Atorvastatin; Ethanol (Control)	-	-	-	-	-	-
Llobet, et al, 2014 [26]	A431; FADU	Cetuximab; Simvastatin; Radiation; Radiation + Simvastatin; Cetuximab + Simvasatin; Radiation + Cetuximab; Radiation + Cetuximab + Simvastatin; DMSO (Control)	-	-	-	-	-	-
Ma et al, 2012 [8]	SCC9; SCC 25; Hela; A549	Lovastatin; Metformin; Gefitinib; Lovastatin + Gefitinib; Ethanol (Control)	24–48–72	0–100 μM Lovastatin; 0–20 mM Metformin; 0–100 μM Gefitinib	√	√	√	1
Mantha et al, 2003 [21]	SCC9; SCC25; MCF-7	Lovastatin; Cisplatin; 5-FU; Paclitaxel; Carboplatin; Oxaliplatin	24–48	0–100 μM Lovastatin; Lovastatin + combination 0–12 μg/mL of each drug	√	√	√	2
Mantha et al, 2005 [11]	SCC9; SCC25; FADU; CAL27; MCF-7	Lovastatin; Pravastatin; Gefitinib; Lovastatin + Gefitinib	24–48–72	0–100 μM Lovastatin	√	√	√	2
Niknejad et al, 2007 [22]	SCC9; SCC 25; Hela	Lovastatin; Lovasatin + Salubrinal	24–48	0–50 μM Lovastatin; 10 μM Lovastatin + 10–75μM Salubrinal	√	√	√	2
Nikejad et al, 2014 [6]	SCC25; Hela; 293T	Lovastatin; Lovastain + Mevalonate; Ethanol (Control)	24–48	0–50 μM Lovastatin; 0–50 μM Lovastatin + 100 μM Mevalonate	√	√	√	1
Pioche- Durieu et al, 2005 [24]	C15; C17	Simvastatin; DMSO (Control)	-	-	-	-	-	-
Takeda et al, 2006 [27]	Tu-167; JMAR	Simvastatin; DMSO (Control)	24–48	0–10 μM Simvastatin	√	√	√	1
Wang et al, 2011 [28]	CCL-30	Statin; Mevalonate; Statin + Mevalonate; Cisplatin; Cisplatin + Statin; Ethanol (Control)	24–48	0–100 μM Statin; 3.5–35 μM Cisplatin	√	√	√	1

Cells: Head and Neck Squamous Cell Carcinoma cell lines, (immortalized or primary cell lines). C: Control. O: Outcomes S: Study (RCT or Comparable baselines). Yes- √”, No “–“. Percentage of cell viability: 1 = 0 to 49% of viable cells; 2 = 50 to 100% of viable cells.

### Risk of bias

We used the GRADE method [18] to assess the quality of the studies. Six studies were categorized as moderate quality [7, 9, 20, 24, 26, 28]. One of these [20] did not show results in all the tested cell lines, so the study could not answer all of our questions. Another study [9] was unclear because we were unable to extract precise data from the graphics as published; however, qualitatively it is clear that the statin has a positive effect on the cell line tested. The articles published by Pioche-Durieu et al. [24] in 2005 and by Islam et al. [7] in 2013 did not completely pertain to the review questions defined in this systematic review; therefore, we considered their usefulness to this work limited. Specifically, the article by Pioche-Durieu et al. [24] did not quantify cell viability, and the papers by Islam et al. [7] and Llobet, et al. [26] did not test cell viability, although they did show that statins regulated the expression of signal transducer and activator of transcription 3 (STAT3) and ERK 1/2 extracellular signal-regulated kinases (ERK 1/2) in HNSCCs. Lastly, the 2011 paper by Wang et al. [28] tested only one cell line and did not compare the results to another cell line (Table 3).

**Table 3:** Judgment of the quality of evidence for intervention.

Authors	GRADE Factors								
	Study design	Study limitation	Inconsistency	Indirectness	Imprecision	Publication bias	Moderate/Large effect size	Dose effect	Overall quality
Dayekh et al, 2014 [23]	With Comparable Baseline	√	√	√	√	√	√	√	++++
Dimitroulakos et al, 2001 [20]	With Comparable Baseline	√	√	X: There are no results to all of the cell lines.	√	√	√	√	+++
Dimitroulakos et al, 2002 [9]	With Comparable Baseline	√	√	Unclear: The graphic results are not viable to analyze.	√	√	√	√	+++
Gabryś et al, 2008 [25]	With Comparable Baseline (*in vitro*) RCT (Animal)	√	√	√	√	√	√	√	++++
Islam et al, 2013 [7]	With Comparable Baseline (*in vitro*) RCT (Animal)	√	√	X: The outcomes considered do not fully answer our questions.	√	√	√	√	+++
Llobet, et al, 2014 [26]	With Comparable Baseline (*in vitro*) RCT (Animal)	√	√	X: The outcomes considered do not fully answer our questions.	√	√	√	√	+++
Ma et al, 2012 [8]	With Comparable Baseline	√	√	√	√	√	√	√	++++
Mantha et al, 2003 [21]	With Comparable Baseline	√	√	√	√	√	√	√	++++
Mantha et al, 2005 [11]	With Comparable Baseline	√	√	√	√	√	√	√	++++
Niknejad et al, 2007 [22]	With Comparable Baseline	√	√	√	√	√	√	√	++++
Nikejad et al, 2014 [6]	With Comparable Baseline	√	√	√	√	√	√	√	++++
Pioche- Durieu et al, 2005 [24]	With Comparable Baseline	√	√	X: The outcomes considered do not fully answer our questions.	√	√	√	√	+++
Takeda et al, 2006 [27]	With Comparable Baseline	√	√	√	√	√	√	√	++++
Wang et al, 2011 [28]	With Comparable Baseline	√	√	√	X: There is just one cell line, with no comparable.	√	√	√	+++

Grade Factors: √, No Serious Limitations; X, Serious Limitations (or not present for moderate/large effects size, dose effect size; dose effect); Unclear, Unable to rate item based on available information. For Overall Quality of Evidence: +, very low; ++, low; +++, moderate; ++++, high.

We also evaluated the evidence regarding the possible clinical application of statins based on the in vitro studies found in this search and classified each paper as either: (1) showing a potential effect following HNSCC treatment; (2) inconclusive and (3) evidence not supportive of using statins to treat HNSCC (Table 1).

### Synthesis of results

#### Cell viability

The cytotoxicity of statins in HNSCC cells lines was evaluated using MTT [6–9, 11, 20–22, 27, 28] and trypan blue assays [25]. Some studies [6, 8, 9, 11, 20, 22, 27, 28] showed that statins used alone are cytotoxic to HNSCC cells and reduced cell viability to less than 50% in a dose dependent manner. Some studies that involved co-administration with another drug showed no significant differences in response [21, 25]. However, when lovastatin was used with gefitinib in SCC9 cells (tongue squamous cell carcinoma), the authors concluded that this combination can enhance cell death to more than 90%, compared with each drug alone [8,11]. The same results were found with lovastatin combined with erlotinib and monensin in SCC9 and SCC25 cells [23]. Co-treatment of CCL-30 (nasopharyngeal carcinoma cells) using cisplatin and statins demonstrated that lovastatin has a significant impact on cell survival. Treating cells with cisplatin (35 μM) and statins (50 μM) together for 24 or 48 hours resulted in 60–90% cytotoxicity (p < 0.05) [24]. Pioche-Durieu et al. [21] found that simvastatin is cytotoxic to NPC cells (nasopharyngeal carcinoma) and it decreases cell viability and changes the cell morphology.

#### Cell cycle regulation and apoptosis

Some studies [6, 11, 20–22, 25, 27] demonstrated accumulation of cells in the G0/G1 phase, as evidenced by flow cytometry assays, when HNSCC cell lines were treated with statins. A 2008 paper by Gabrys et al. [25] demonstrated that 55% of FaDu cells (pharyngeal squamous cell carcinoma) were in the G0/G1 phase following treatment with 25 μM lovastatin for 48 hours, but this was not significantly different from the control (40%) or cells treated with 25 μM lovastatin for 48 hours and 4 Gy (Grays) (55%). Takeda et al. [27] in 2006 showed the dose-dependent inhibitory effects of different concentrations of simvastatin (0–10 μM) on the cell growth and cell cycle of oral squamous cell carcinoma cell lines (JMAR and Tu167) after 48 hours. They showed that statin can control the cell cycle and apoptotic signaling, and there was a statistically significant difference between the control and treated cells (p < 0.01). In 2003, Mantha et al. [21] found that lovastatin induced apoptosis in SCC25 cells (tongue squamous cell carcinoma), and the results were significant at doses of both 10 μM (20.4% and 50 μM (34%) after 48 hours of treatment. The cells in G0/G1 were 68.4% of the total 48 hours after treatment with a 10 μM dose and 74.2% after treatment with a 50 μM dose. No significant differences in response were evident between both lovastatin and chemotherapeutic treatments alone or in combinations. In 2005, Mantha et al. [11] confirmed this in an additional paper.

#### Regulation of protein expression level

Islam et al. [7] in 2013 demonstrated in vitro (using UM-SCC-1 squamous cell carcinoma cells derived from floor of the mouth and UM-SCC-47 squamous cell carcinoma cells derived from floor of the tongue) and also in vivo that atorvastatin treatment reduces the activity of RhoC (a GTPase belonging to Ras superfamily, which is over-expressed in a wide range of invasive carcinomas) to 48% of control in UM-SCC-1 cells and to 52% in UM SCC-47 cells. They showed that RhoC activates the ERK1/2 and STAT3 pathways by regulating their phosphorylation in HNSCC. In vivo animal experiments showed an inhibition of angiogenesis and lung metastasis after atorvastatin therapy (p < 0.05).

Ma et al. [8] in 2012 evaluated the effect of lovastatin on the activity of the liver kinase B1 (LKB1)/AMP-activated protein kinase (AMPK) pathway. An MTT assay was carried out using wild type LKB1^+/+^ murine embryonic fibroblasts (MEFs) as a control, which was compared to LKB1^-/-^ treated with a range of lovastatin concentrations of up to 25 μM for 48 and 72 hours. Their data showed that LKB1^+/+^ cells (20% cell viability after 25 μM dose) were significantly more sensitive than LKB1^-/-^ cells (90% cell viability in 25 μM) after 48 hours. After 72 hours, there was no appreciable difference in the cytotoxicity in these two sets of MEFs (20% cell viability after 25 μM), and both cell lines responded in a dose dependent manner. Lovastatin-induced apoptosis is regulated in part by the inhibition of the EGFR. Western blot analysis of SCC25 cells treated for 24 hours showed that lovastatin regulates downstream targets of EGFR (pAKT), the Integrated Stress Response (ISR), and cell cycle regulators, cyclin D1 and p21, and eukaryotic translation initiation factor 4E-binding protein 1 (4EBP1).

In 2007, Niknejad, Morley, and Dimitroulakos [22] used a cell viability assay to compare the effect of lovastatin in CHOP (a member of the activating transcription factor (ATF) family)^-/-^ and CHOP ^+/+^ MEFs after 0–25 μM doses and 48 hours. Statistically significant differences were found according to a one-tailed t-test (p < 0.05). A significant difference was observed in the populations of sub G1 apoptotic cells in CHOP-^/-^ and CHOP ^+/+^ MEFs that were treated with 10 μM doses for 24 and 48 hours (p < 0.05).

The work of Takeda et al. [27], in 2006, showed that 5–10 μM doses of simvastatin could induce upregulation of active caspase-3 expression after a 48-hour treatment. The influence of mevalonate on morphology, cell viability, and active caspase-3 expression were demonstrated after the treatment with simvastatin.

Llobet, et al, in 2014 [26] demonstrated that simvastatin, in treatment combined with radiation and cetuximab, can decrease wound healing, cell proliferation, slowed the growth of FADU xenografts and induce apoptosis. Pro caspase-3 expression was demonstrated after the treatment with simvastatin (p< 0.05 compared to radiation plus cetuximab without simvastatin). They also investigated whether simvastatin could affect crucial cellular signaling pathways involved in the malignat phenotype of cancers. They found the additions of simvastatin to radiation did not modify phosphorylated levels of EGFR, but they showed cetuximab had an inhibitory effect on the radiation induced p-EGFR (phosphorylation of EGFR), indicating that simvastatin had a little effect on EGFR. Simvastatin has a weak effect on the activation of phosphorylated AKT and phosphorylated STAT3.

Dayekh et al, [23] used monensin, lovastatin and erlotinib alone and in combination to treat SCC9, SCC25 and GM-38 (normal lung fibroblasts) cells. They confirmed the ability of monensin to potentiate the cytotoxicity of erlotinib and lovastatin and showed that combination of 5μmol/L of monensin with 10μmol/L erotinib for 24 hours have 38.7% of apoptosis in SCC25 cells. They did not use lovastatin on flow-cytometry experiments. With the western blots assay they found that monensin can inhibts phosphorylation status of EGFR and its downstream targets AKT and ERK proteins were assessed in SCC9 cells treated with 10μmol/L lovastatin for 24 hours. Both 10μmol/L lovasatin and 1μmol/L monensin for 24 hours treatments induced approximately a 50% inhibition of EGF- treated SCC9 cell with respect to pEGFR and its downstream targets pAKT and pERK (phosphorylated extracellular signal-regulated kinases).

### Risk of bias across studies

The studies selected for this analysis were considered heterogenous, and they did not have compatible data that would allow a meta-analysis. In addition to the non-comparability of the results of each study, a meta-analysis could not be conducted due to lack of clinical studies on this subject.

## Discussion

### Summary of evidence

The incidence of HNSCC has been on the increase and has become an important issue [29]. Oral cancer is a serious and growing problem worldwide and new therapies are emerging [30,31]. The major risk factors for these cancers are smoking and alcohol, although, infection with human papillomavirus (HPV) virus–especially subtype 16 –is also a risk factor, particularly for tonsil carcinoma [32]. The successful treatment of HNSCC is a challenge. Conventional approaches have allowed HNSCC to be controlled and improved the overall survival rate but only in the early stages of the disease [33]. Advanced tumors in the recurrent and distant metastases stages are often treated with combination therapy involving surgical resection followed by radiotherapy and sometimes chemotherapy [34]. All of these treatments are cytotoxic and are associated with many adverse effects that reduce the quality of life of patients. Therefore, new perspectives and therapeutic approaches are needed for successful and less cytotoxic HNSCC treatments [29].

The statins are a remarkably effective class of drugs that lower cholesterol levels in blood and reduce the frequency of heart attacks [1]. No major adverse effects resulting from the lowering of cholesterol have been noted in any studies. The remarkable safety of statins is derived from their unique mechanism of action. The statins are the largest selling class of drugs currently taken by patients worldwide [1].

There has been a growing interest in statins because of their anticancer effects [5]. A systematic review and meta-analysis performed by Dale et al. [35], in 2006, showed statins have a neutral effect on cancer and cancer death risk in randomized controlled trials [35]. They found that no type of cancer was affected by statin use, but they did not report any studies involving HNSCC. We only found a phase one clinical trial that involved prolonged administration of lovastatin to patients with recurrent or metastatic squamous cell carcinoma of the head and neck [10]. The authors demonstrated that lovastatin stabilized the disease in 23% of the treated HNSCCs, and the authors concluded that further clinical evaluations of statins were required to evaluate their potential as part of combination therapy [10].

Furthermore, several studies have tested statin-derived drugs (lovastatin, simvastatin, atorvastatin, and pravastatin) on HNSCC cell lines [7, 8, 11, 20, 22, 27]. In this systematic review, we studied the effect of statins on head and neck cancer and found 14 relevant in vitro studies [6–9, 11, 20–28]. These in vitro studies supported the idea that statins have potential as a therapy for HNSCC. To evaluate the effect of statins on HNSCC cells, all the authors that used MTT assays or trypan blue assays [8, 11, 20–25] demonstrated that statins alone could effectively and dose dependently kill more than 50% of the cells and inhibit proliferation (Table 2). The effect of statins on HNSCC cells was also evaluated in combination with radiation [25] or with other chemotherapeutics (cisplatin, 5-fluorouracil, paclitaxel, carboplatin, and oxaplatin) [21], but in these cases, the statins had no significant differences. However, when a combination of gefitinib and lovastatin was tested in SCC9 cells, cell death was enhanced by more than 90% when compared to each drug alone [8, 11]. The same results were found by Dayekh et al. [23] when used lovastatin combined with monensin and erlotinib.

Llobet et al., in 2014, [26] showed that simvastatin might enhance antitumor response when in combination with radiation and cetuximab. They concluded that simvastatin can decrease wound healing, cell proliferation and colony formation and can induce apoptosis on FaDu cells.

Derivatives of the statin drugs are potential inhibitors of HMG-CoA reductase, an enzyme derived from the mevalonate pathway [6, 11, 36]. The viability of malignant cells is dependent on the final products of the mevalonate pathway that includes *de novo* cholesterol and isoprenoids [5, 6, 36]. Many of these products are used in cell proliferation and also required for critical cellular functions such as maintenance of cell membrane integrity, signaling, protein synthesis, and cell cycle progression. Interruption of this process in malignant cells results in inhibition of tumor growth and metastasis [9]. Simvastatin can regulate the expression of phosphorylated forms of ERK1\2 (extracellular-signal-regulated kinases) and the expression of cell cycle regulators, such as p21 and p27 in HNSCC cells [6, 27]. In vitro, atorvastatin treatment significantly reduces the active form of RhoC. In addition, atorvastatin induces a significant decrease in phosphorylated forms of ERK1/2 and STAT3, and reduces cell motility, invasion, proliferation, and colony formation. These in vitro results provide evidence that statin treatment can be a useful therapeutic for HNSCC. Using a murine flank model implanted with UM-SCC-47 (tongue squamous cells carcinoma) atorvastatin was shown to decrease tumor growth [7]. In contrast, Llobet et al. found that simvastatin had no effects on the levels of total EGFR, ERK1/2, AKT, and STAT3 when they used in combination with radiation and cetuximab to treat FaDu cells [26].

Statins have been shown to inhibit the mevalonate, PI3K/AKT, and mTOR pathways in oral cancer [6, 8, 29]. Additionally, they can inhibit the function of EGFR and induce the LKB1/AMPK pathway that is activated upon ATP depletion during metabolic stress [6, 8, 29]. This drug has the ability to influence different routes of tumor induction mediated by metabolic stresses (ISR), ATF3 expression, and CHOP, which regulates apoptosis of HNSCC cells [6, 8]. Salubrinal, an agent that is able to prolong the activity of stress-induced ATF3 expression, in combination with lovastatin could enhance cytotoxicity and induce apoptosis. These authors suggested that ATF3 inducers, in combination with agents such as lovastatin or salubrinal, could be clinically useful [6].

The tumor suppressor protein p21 has been shown to inhibit tumor proliferation and cell-cycle progression, which may contribute to tumor suppression [37]. One study has shown that simvastatin can regulate the expression of proteins related to cell cycle progression such as p21 and p27 in HNSCC cells [27]. Another study has shown that in HNSCC cells, lovastatin treatments alone can upregulate the expression of p21, while irradiation alone cannot [25]. It has been suggested that lovastatin is responsible for the upregulation of the cell cycle inhibitor p21, leading to G1 arrest [1]. The studies included in this review confirm this statement. Lovastatin treatment in oral cancer cells demonstrated a pronounced pre-G1 apoptotic peak, and simvastatin induced a dose-dependent accumulation of G1 phase cells and decreased the total S-phase population in an oral squamous cell carcinoma [6, 21, 27]. Gabry’s et al. [25] confirmed in 2008 that lovastatin alone arrested HNSCC cells in G0/G1 phase and induced apoptosis.

In summary, this is the first systematic review of the effects of statins on HNSCC treatment and the first review to present evidence of a positive association between decrease of HNSCC cell viability and statin using in vitro assays. Although, in 2006, Dale et al. [35] performed a systematic review and meta-analysis of the relationship between statins and cancer risk in cancer patients who use statin drugs; they did not analyze head and neck cancer patients. Currently available evidence suggests that statins could be used to treat HNSCC, although this evidence comes from only a few studies. These studies prove that statins could inhibit the growth, invasion, and metastasis of tumor cells and reduce cellular proliferation and differentiation. In addition, statins regulate the cell cycle resulting in the inhibition of the proliferative effects of the malignant cells [6–9]. Furthermore, statins can induce apoptosis especially in squamous cell carcinoma [11, 21, 23–25, 27, 28].

These in vitro studies [6–9, 11, 20–27] confirmed the anticancer effects of the statin drugs and the potential promise of statin compounds as adjunct to standard therapies available for HNSCC [7]. It may represent a novel therapeutic approach in HNSCC. However, further clinical evaluation of statins should evaluate their potential as part of a combination of a target therapy, combining modality approach or as Phase II cytostatic agents [10].

### Limitations

Some methodological limitations of this review should be considered. We found only one clinical study that used statins in patients with HNSCC. Of 153 papers, we selected 14 studies using our inclusion criteria and they were in vitro and in vivo animal studies. For the quality assessment of in vitro studies, we did not use any standard assessment for basic studies; however, we defined a method to assess the quality of all articles. Thereby, we have classified in vitro studies using comparable baselines, comparing them to the RCT studies published by Xiao et al. in 2013 [19] using the GRADE method [18]. Furthermore, we have categorized six studies as moderate quality [7, 9, 20, 22, 26, 28] because they did not fully answer the review questions defined in this systematic review. The insufficient reporting of clinical studies prohibits clinical analysis. We suggest future research to eliminate these limitations.

## Conclusions

This study demonstrated that statins have a significant effect on HNSCC cell lines with respect to cell viability, cell cycle, cell death, and the regulation of protein expression levels involved in pathways of carcinogenesis, which corroborates with the potential in vitro anti-tumor effects. Though there are few studies on the topic, currently available evidence suggests that statins are potentially useful for HNSCC treatment, and their use requires further clinical analysis.

## Supporting Information

S1 PRISMA Checklist PRISMA Checklist. (DOC)

S1 Table Database Search. (DOC)

S2 Table Excluded articles and reasons for exclusion (n = 4). (DOC)
