# Supplementary material for: In vitro Anti-Tumor Effects of Statins on Head and Neck Squamous Cell Carcinoma: A Systematic Review
Source: PLoS One. 2015 Jun 22;10(6):e0130476. doi: 10.1371/journal.pone.0130476 (PMC4476585; doi:10.1371/journal.pone.0130476)
Supplement: S1 Table — (DOC) [file pone.0130476.s002.doc]

**S1 Table.** Database Search

| **Database** | **Search** |
| --- | --- |
| **Cochrane (June 30, 2014 – update May, 10, 2015)** | # 1 ‘Statin and head and neck cancer’; #2 ‘Statin and squamous cell carcinoma; #3 Statin and squamous cell cancer; #1 or #2 or #3 |
| **EMBASE (June 30, 2014 – update May, 10, 2015)** | #1 Statin.mp.; #2 oral cancer.mp.; #3 oral carcinoma.mp.; #4 head carcinoma.mp.; #5 neck carcinoma.mp.; #6 head cancer.mp.; #7 neck cancer.mp., squamous cell carcinoma.mp; #2 or #3 or #4 or #5 or #6 or #7 or #8; #1 and #9 [mp=title, abstract, subject headings, heading word, drug trade name, original title, device manufacturer, drug manufacturer, device trade name, keyword] |
| **LILACS (June 30, 2014 – update May, 10, 2015)** | #1 estatinas.mp.; #2 cancer.mp.; [mp=title, abstract, subject headings, heading word, drug trade name, original title, device manufacturer, drug manufacturer, device trade name, keyword] #1 and #2 |
| **MEDLINE (June 30, 2014– update May, 10, 2015)** | #1 Statin.mp.; #2 Cancer.mp.; #3 Carcinoma.mp.; #4 Oral .mp.; [mp=title, abstract, subject headings, heading word, drug trade name, original title, device manufacturer, drug manufacturer, device trade name, keyword] #2 or #3 or #4; #1 and #5 |
| **PubMed (June 30, 2014 – update May, 10, 2015)** | #1 ‘Statin’; #2 ‘Neck cancer’; #3 ‘Head and neck cancer’; #4 ‘Squamous cell carcinoma’; #5 ‘Oral Squamous Cell Carcinoma; #6 - (#2 or #3 or #4 or #5); (#1) and #6 |
