# Supplementary material for: In vitro Anti-Tumor Effects of Statins on Head and Neck Squamous Cell Carcinoma: A Systematic Review
Source: PLoS One. 2015 Jun 22;10(6):e0130476. doi: 10.1371/journal.pone.0130476 (PMC4476585; doi:10.1371/journal.pone.0130476)
Supplement: S2 Table — (DOC) [file pone.0130476.s003.doc]

**S2 Table.** Excluded articles and reasons for exclusion (n=4).

| **Author, Year** | **Reason for exclusion** |
| --- | --- |
| Avgoustidis et al, 2012 ^1^ | 1 |
| Deng et al, 2011 ^2^ | 1 |
| Knox et al, 2005 ^3^ | 3 |
| Miglierini et al, 2013 ^4^ | 2 |

1- Articles with different target condition, such as studies that did not use statin to treat cancer or did not verify the association between statin and HNSCC (n=2), 2- Reviews of the literature, letters, personal opinions, conference abstracts and book chapters (n=1) and 3- Clinical studies (n=1).

REFERENCES

1. Avgoustidis D, Nisyrios T, Nkenke E, Lijnen R, Ragos V, Perrea D, Donta I, Vaena A, Yapijakis C, Vairaktaris E. Oral carcinogenesis is not achieved in different carcinogen-treated PAI-1 transgenic and wild-type mouse models. In Vivo 2012; 26(6): 1001-5.
2. [Deng YT](http://www.ncbi.nlm.nih.gov/pubmed?term=Deng YT%5BAuthor%5D&cauthor=true&cauthor_uid=21317023), [Chang JZ](http://www.ncbi.nlm.nih.gov/pubmed?term=Chang JZ%5BAuthor%5D&cauthor=true&cauthor_uid=21317023), [Yeh CC](http://www.ncbi.nlm.nih.gov/pubmed?term=Yeh CC%5BAuthor%5D&cauthor=true&cauthor_uid=21317023), [Cheng SJ](http://www.ncbi.nlm.nih.gov/pubmed?term=Cheng SJ%5BAuthor%5D&cauthor=true&cauthor_uid=21317023), [Kuo MY](http://www.ncbi.nlm.nih.gov/pubmed?term=Kuo MY%5BAuthor%5D&cauthor=true&cauthor_uid=21317023). Arecoline stimulated Cyr61 production in human gingival epithelial cells: inhibition by lovastatin. [Oral Oncol](http://www.ncbi.nlm.nih.gov/pubmed/?term=oral+oncology+47+256-261) 2011; 47(4):256-61.
3. [Knox JJ](http://www.ncbi.nlm.nih.gov/pubmed?term=Knox JJ%5BAuthor%5D&cauthor=true&cauthor_uid=15737556), [Siu LL](http://www.ncbi.nlm.nih.gov/pubmed?term=Siu LL%5BAuthor%5D&cauthor=true&cauthor_uid=15737556), [Chen E](http://www.ncbi.nlm.nih.gov/pubmed?term=Chen E%5BAuthor%5D&cauthor=true&cauthor_uid=15737556), [Dimitroulakos J](http://www.ncbi.nlm.nih.gov/pubmed?term=Dimitroulakos J%5BAuthor%5D&cauthor=true&cauthor_uid=15737556), [Kamel-Reid S](http://www.ncbi.nlm.nih.gov/pubmed?term=Kamel-Reid S%5BAuthor%5D&cauthor=true&cauthor_uid=15737556), [Moore MJ](http://www.ncbi.nlm.nih.gov/pubmed?term=Moore MJ%5BAuthor%5D&cauthor=true&cauthor_uid=15737556), [Chin S](http://www.ncbi.nlm.nih.gov/pubmed?term=Chin S%5BAuthor%5D&cauthor=true&cauthor_uid=15737556), [Irish J](http://www.ncbi.nlm.nih.gov/pubmed?term=Irish J%5BAuthor%5D&cauthor=true&cauthor_uid=15737556), [LaFramboise S](http://www.ncbi.nlm.nih.gov/pubmed?term=LaFramboise S%5BAuthor%5D&cauthor=true&cauthor_uid=15737556), [Oza AM](http://www.ncbi.nlm.nih.gov/pubmed?term=Oza AM%5BAuthor%5D&cauthor=true&cauthor_uid=15737556). A Phase I trial of prolonged administration of lovastatin in patients with recurrent or metastatic squamous cell carcinoma of the head and neck or of the cervix. [Eur J Cancer](http://www.ncbi.nlm.nih.gov/pubmed/?term=a+phase+I+trial+of+prolongued+administration+of+lovastatin+in+patients) 2005; 41 (4): 523.
4. Miglierini, P, Rave-Frank M, Wolff H, Pradier O. In vitro effects of the combination of Lovastatin and irradiation on human cell lines. Conference: 19. Jahreskongress der Deutschen Gesellscraft fur Radioonkologie, DEGRO 2013; 189. Journal: Conference Abstract.
